# Students' Evaluation of a Team-based Course on Research and Publication Ethics: Attitude Change in Medical School Graduate Students

**DOI:** 10.3352/jeehp.2008.5.3

**Published:** 2008-12-22

**Authors:** Soo Young Kim

**Affiliations:** Department of Family Medicine, College of Medicine, Hallym University, and Kangdong Sacred Heart Hospital, Seoul, Korea.

**Keywords:** Research and Publication Ethics, Team-based Learning, Graduate Students, Survey, Course Evaluation

## Abstract

In response to a growing need for students to appreciate ethical issues in medical research and publication, a brief team-based learning (TBL) course was presented to graduate students in the medical school of Hallym University in October and November 2007. To gather information as a basis for improving the course, questionnaires were distributed to 19 students and the feedback was evaluated. The questionnaire consisted of four categories: general course content (7 items), changes in attitudes toward research and publication ethics (6 items), the TBL format (6 items), and an open-ended question about the class (1 item). The most positive response had to do with the importance of the material. Students reported that their knowledge about ethical issues increased, and they expressed satisfaction regarding the communication with their tutors within the TBL format. Most students showed positive responses to the subject as well as to TBL. Since this was the first trial offering of this material in the graduate program at this medical school, it may have been novel to the students. The attitude change and the knowledge acquisition reported by students reflect a very positive outcome of this class. After adjustments to improve weaknesses, such as the short time allocation and students' lack of prior background, the outcomes of this TBL course on research and publication ethics provide a good basis for its continuation.

## INTRODUCTION

Since February 2007, a lecture and team-based learning (TBL) module on research and publication ethics has been offered for new faculty as part of the workshop for incoming faculty at the College of Medicine, Hallym University. During the workshop, it became apparent that young faculty members rarely had an opportunity to learn about these issues during their training period as residents or fellows. Incredibly, the most frequently occurring issue was the definition of authorship. Once this problem was identified, the TBL course continued to be offered for incoming faculty. Discussions among tutors led to the observation that it would be better for graduate students, who are deeply involved in research and publication, to be exposed to this information. Therefore, during a graduate course titled Methods of Medical Research, 4 hr were allocated to the ethics of research and publication, 2 hr on each of two separate days with a 14-day interval between them, in October and November 2007. In this article, the content of the TBL ethics course will be described and students' evaluations will be examined as a basis for improving the class.

## MATERIALS AND METHODS

The content of the course focused on basic issues involved in research and publication ethics and was developed by four tutors, including the author, who are faculty members of the College of Medicine, Hallym University. The tutors discussed the contents via the Internet and agreed on the topics to be addressed in the graduate course. The textbook for the course was also written by the four tutors.

To gather feedback from the students, a survey using a series of 5-point Likert-scale questions was administered at the end of the 4-hr course. Nineteen students responded to the survey. Some students were physicians working in the hospital or local clinics, and the others were full-time students in basic medicine. The questionnaire consisted of four categories: general course content (7 items), attitudes toward research and publication ethics (6 items), the TBL format (6 items), and an open-ended question about the course (1 item). The items in each category are described in Table 1, 2, and 3. The answers to the open-ended question, when students could discuss the course freely, are summarized below.

## RESULTS

The nine topics covered in the textbook were as follows: Introduction to Research Ethics, Research Misconduct, Ethics in Clinical Research, Conflict of Interest, Copyright Protection, Plagiarism, Authorship, Duplicate Publication, and Publication Ethics. The textbook was distributed to students 2 weeks prior to the first ethics segment of the course. During the TBL, assessments of individual and group readiness and of the application of course material were conducted. Assessment items consisted of real cases that have occurred in Korea, providing students with an opportunity to seek solutions in situations that might have some personal meaning [1]. The results are summarized in Table 1, 2, and 3. Mean and median responses to each item and percentage of strongly positive responses (≥4) to each item are shown.

Results indicated that students were generally satisfied with the contents of the course. The most positive response affirmed the necessity of learning the material covered in the class. Students also reported satisfaction with the textbook and with the level of information covered in the book. In contrast, students were relatively dissatisfied with the fast pace of the course, the amount of material covered, and the time allocated for the material, probably due to the extensive amount of information covered in the textbook (Table 1). Students' responses also reflected positive attitudes about research and publication ethics. This finding may also be taken as a positive comment about the students. Some students expressed concern as to whether their preceptor had been involved in ethical misconduct (Table 2). They were generally satisfied with the communication with a tutor provided by the TBL format. Most students showed positive responses to the subject matter as well as to the TBL format. Students reported an increase in workload due to preparation for the class and to the TBL format. In addition, class participation itself may have been viewed as an increased burden, as students were expected to participate in group discussion (Table 3). Responses to the open-ended question can be summarized as follows: It was difficult to understand research ethics; The time allotted for the class was too short; I gained new knowledge; The TBL format was useful; A system to preserve research and publication ethics is necessary; Concerns that arose while writing the class paper will arise again; This course should also be implemented in the residency program at the hospital.

## DISCUSSION

Keeping up with the rapid pace of the course may be too hard for some students when it is offered in such a short period, and some concepts might be difficult for students at this level. Some students reported that preparing for and participating in this course involved a heavier workload than other courses. This observation may in part reflect a lack of familiarity with TBL classes. TBL has been applied to very few classes in the graduate school of the College of Medicine, Hallym University. Although some students complained of difficulties in preparing for and participating in the class and problems in understanding the contents, most students were very satisfied not only with the contents but also with the TBL format of this course.

Finding instances of the implementation of TBL in courses on research and/or publication ethics at the graduate level is difficult, although numerous instances of TBL can be found in undergraduate medical school courses. A study on TBL in an anatomy and embryology course in the United States suggested that TBL may be most beneficial for at-risk students, as this format ensures that they receive encouragement to study consistently and are provided regular feedback on their preparedness and given the opportunity to develop higher reasoning skills [2]. TBL has been found to be an effective active teaching method for teaching dermatopathology in Korea [3]. Another report indicated that a team-based continuing medical education course led to significant gains in participants' knowledge in Germany [4]. Since the need for knowledge of and for positive attitudes toward medical practice are essential in medical research [5], TBL may be a good learning tool for addressing research and publication ethics in the classroom and in the field. To ensure good ethical practices among the new generation of medical doctors, the TBL course on ethical issues should be implemented in the residency training program of every training hospital as well as in graduate programs.

## Figures and Tables

**Table 1:**
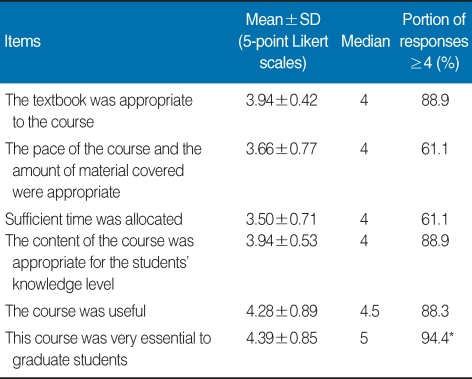
Graduate students' responses to six items addressing general satisfaction with the course on research and publication ethics, college of medicine, Hallym university, 2007

**Table 2:**
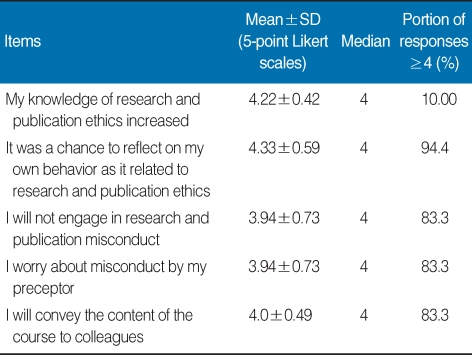
Graduate students' responses to five items measuring attitudes toward research and publication ethics following the course on research and publication ethics, college of medicine, Hallym university, 2007

**Table 3:**
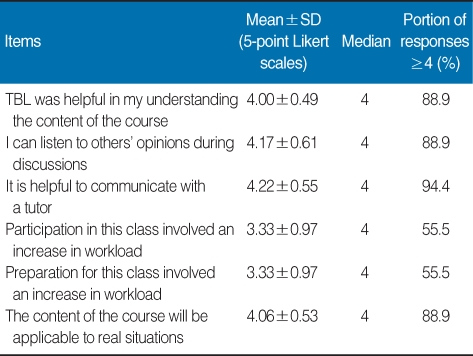
Graduate students' responses to six items related to TBL in the research and publication ethics course, college of medicine, Hallym university, 2007
